# Implementation of a high fidelity simulation based training program for physicians of children requiring long term invasive home ventilation: a study by ISPAT team

**DOI:** 10.3389/fped.2024.1325582

**Published:** 2024-02-01

**Authors:** Nilay Bas Ikizoglu, Emine Atag, Pinar Ergenekon, Yasemin Gokdemir, Zeynep Seda Uyan, Saniye Girit, Ayse Ayzit Kilinc Sakalli, Ela Erdem Eralp, Erkan Cakir, Feray Guven, Mehmet Emin Aksoy, Bulent Karadag, Fazilet Karakoc, Sedat Oktem

**Affiliations:** ^1^ Division of Pediatric Pulmonology, Istanbul Faculty of Medicine, Istanbul University, Istanbul, Turkiye; ^2^ Division of Pediatric Pulmonology, School of Medicine, Maltepe University, Istanbul, Turkiye; ^3^ Division of Pediatric Pulmonology, School of Medicine, Marmara University, Istanbul, Turkiye; ^4^ Division of Pediatric Pulmonology, School of Medicine, Koc University, Istanbul, Turkiye; ^5^ Division of Pediatric Pulmonology, Faculty of Medicine, Medeniyet University, Istanbul, Turkiye; ^6^ Division of Pediatric Pulmonology, Cerrahpasa Faculty of Medicine, Istanbul-Cerrahpasa University, Istanbul, Turkiye; ^7^ Division of Pediatric Pulmonology, Faculty of Medicine, Istinye University, Istanbul, Turkiye; ^8^ Center of Advanced Simulation and Education (CASE), Acibadem Mehmet Ali Aydinlar University, Istanbul, Turkiye; ^9^ Division of Pediatric Pulmonology, School of Medicine, Medipol University, Istanbul, Turkiye

**Keywords:** long term ventilation, home ventilation, home care, mechanical ventilation, children, high fidelity simulation, tracheostomy

## Abstract

**Introduction:**

The number of children requiring long-term invasive home ventilation (LTIHV) has increased worldwide in recent decades. The training of physicians caring for these children is crucial since they are at high risk for complications and adverse events. This study aimed to assess the efficacy of a comprehensive high-fidelity simulation-based training program for physicians caring for children on LTIHV.

**Methods:**

A multimodal training program for tracheostomy and ventilator management was prepared by ISPAT (IStanbul PAediatric Tracheostomy) team. Participants were subjected to theoretical and practical pre-tests which evaluated their knowledge levels and skills for care, follow-up, and treatment of children on LTIHV. Following the theoretical education and hands-on training session with a simulation model, theoretical and practical post-tests were performed.

**Results:**

Forty-three physicians from 7 tertiary pediatric clinics in Istanbul were enrolled in the training program. Seventy percent of them had never received standardized training programs about patients on home ventilation previously. The total number of correct answers from the participants significantly improved after the theoretical training (*p* < 0.001). The number of participants who performed the steps correctly also significantly increased following the hands-on training session (*p* < 0.001). All of the 43 participants who responded rated the course overall as good or excellent.

**Conclusion:**

The knowledge and skills of clinicians caring for children on LTIHV can be enhanced through a comprehensive training program consisting of theoretical training combined with hands-on training in a simulation laboratory.

## 1 Introduction

The number of children who are discharged home on long term invasive home ventilation (LTIHV) has been increasing during the recent years. This is due to several factors including advances in technology and medical care, increasing clinical experience and widening of indications for initiating home ventilation. Children dependent on home ventilation are a diverse group of patients with various underlying etiologies including neuromuscular diseases, chronic lung diseases, airway problems and respiratory control disorders. They often have significant associated comorbidities (1–5).

Patients on LTIHV require complex medical care and technological support, they are managed by multidisciplinary teams of physicians including pediatric pulmonologists, intensive care physicians, pediatricians, pediatric nurses, respiratory therapists, otorhinolaryngologists and pediatric surgeons. These children have high rate of hospital readmissions, unplanned emergency visits and are at high risk for complications and death (5, 6). Death is mostly due to progression of underlying disease and associated comorbidities, however tracheostomy complications including accidental decannulation, mucus plugging, and ventilator failure may also cause death and may be preventable (6, 7).

In order to provide optimal care and decrease morbidity and mortality in these patients, all the members of the health care team and caregivers must be well trained to perform routine care, must have skills for tracheostomy care and ventilator management, must be able to recognize and troubleshoot problems, and must respond effectively to emergency situations. The deficiency in education results in low levels of confidence and significant knowledge and skill deficits in many areas of care, potentially limiting physicians' ability to effectively care for patients with tracheostomies on LTIHV (8–14).

In recent years, high fidelity simulation (HFS) training has been utilized increasingly in education of health care providers, because it provides realistic scenarios that allow participants to experience critical situations, to practice their skills, to enhance their knowledge and self confidence and to get feedback without compromising patient safety (15).

The aim of this study was to assess the efficacy of a comprehensive HFS based training program for physicians who manage children on LTIHV. We hypothesized that, a HFS based educational program would increase knowledge of tracheostomy care and ventilator management and facilitate achievement of skills for recognizing and managing tracheostomy and ventilator related emergencies.

## 2 Materials and methods

### 2.1 Preparation for the study

We developed a multimodal training course for tracheostomy and ventilator management consisting of a theoretical part with educational slides and instructional videos, hands-on practice with HFS scenarios of tracheostomy and ventilator related emergencies and theoretical and practical tests. The educational materials, theoretical and practical tests were prepared by ISPAT (IStanbul PAediatric Tracheostomy) team, a multidisciplinary group of specialists consisting of pediatric pulmonologists, pediatric gastroenterologists, otorhinolaryngologists, pediatric surgeon, intensive care specialist, speech/swallowing therapist, physical therapist, dentist, and family representative (16). The HFS scenarios of tracheostomy and ventilator emergencies were reviewed and refined by a certified HFS educator.

Pediatric pulmonologists, pediatric pulmonology fellows, pediatricians, pediatrics residents and pediatric intensive care fellows who take part in the care of children on LTIHV were invited to the study. The study was conducted in accordance with the World Medical Association Declaration of Helsinki. The study protocol was approved by the Ethics Committee of Marmara University, School of Medicine (reference number 1,317). Written informed consent were obtained from all participants.

### 2.2 Baseline assessment

The study was conducted at Acibadem Mehmet Ali Aydınlar University, Center of Advanced Simulation and Education Laboratory between September 2021 and March 2022.

At the beginning of the training course, a pre-test was performed consisting of 12 questions about demographic characteristics of participants identifying their residency type and their previous experiences and training regarding tracheostomy and ventilator management and cardiopulmonary resuscitation. The following part comprised of an objective test with 34 multi-choice questions assessing the knowledge level of participants about tracheostomy and long term invasive ventilation indications and complications, tracheostomy suctioning and cannula change, ventilator modes and ventilator alarms, tracheostomy and ventilator related emergencies, basic life support. Ten questions of the test regarding signs of respiratory distress, basic life support application, contents of the emergency bag, knowledge of tracheostomy suctioning, duration of suctioning, tracheostomy cannula size, frequency of tracheostomy cannula change, indications for invasive ventilation, ventilator equipment and ventilation modes were determined as essential questions.

After the theoretical pre-test, participants were led to the simulation room to familiarize them with the HFS experience. An investigator provided information about the props in the room and the features of the mannequin. The high fidelity mannequin (PediaSIM ECS®, CAE Healthcare, QC Canada) resembles a five year old child and has a tracheostoma that standard tracheostomy cannulas can be fitted and can be connected to a ventilator. The breathing pattern, eye opening and vital signs including respiratory rate, heart rate, oxygen saturation, blood pressure can be altered in response to interventions.

After the familiarization process, each participant was subjected to a practical pre-test consisting of two scenarios: (1) high pressure alarm on ventilator caused by mucus plugging of tracheostomy cannula. (2) low presssure alarm on ventilator caused by a leak in the ventilator circuit. Participants' practical skills were evaluated by a pediatric pulmonologist using a standardized checklist of appropriate interventions. The checklist of the first scenario involved 22 steps and four of these steps (checking the child, making a decision to aspirate, suctioning the cannula, changing the cannula) were determined essential. The checklist of the second scenario involved 12 steps and four of these steps (checking the child, connecting the ventilator to the test lung, checking the ventilator circuit and identifying the leak, changing the ventilator circuit) were determined essential.

### 2.3 Intervention

The study intervention was a 3 h simulation based course which included a 90 min interactive lecture and a 90 min hands-on training session in the simulation lab. The lecture was given by a senior pediatric pulmonologist and involved information about the indications and complications of tracheostomy and long term invasive and non-invasive ventilation, causes of morbidity and mortality in patients on LTIHV, types of tracheostomy cannulas, suctioning and changing of tracheostomy cannulas, ventilator equipment and maintenance, ventilator modes and alarms, tracheostomy and ventilator related emergencies and cardiopulmonary resuscitation. The lecture also included educational videos on tracheostomy care, tracheostomy and ventilator related emergencies and cardiopulmonary resuscitation recorded by ISPAT team.

During hands-on training session, the participants worked in teams of two through four scenarios on the high fidelity patient simulator: high pressure alarm on the ventilator due to obstruction of tracheostomy cannula, malfunction of home ventilator that required change to the spare ventilator, low pressure alarm due to a leak in the ventilator circuit and desaturation event resulting in bradycardia that necessitated cardiopulmonary resuscitation. Two instructors observed the participants and provided feedback on the scenarios and practical performance of the participants.

### 2.4 Assessment after the intervention

After the hands-on training session, the objective multi-choice test with 34 questions (the same test as the pre-test) and practical post-test (the same two scenarios as the pre-test) were applied to the participants. Practical post-test was followed by a debriefing provided by the instructors using checklists of practical tests.

**Figure 1:**
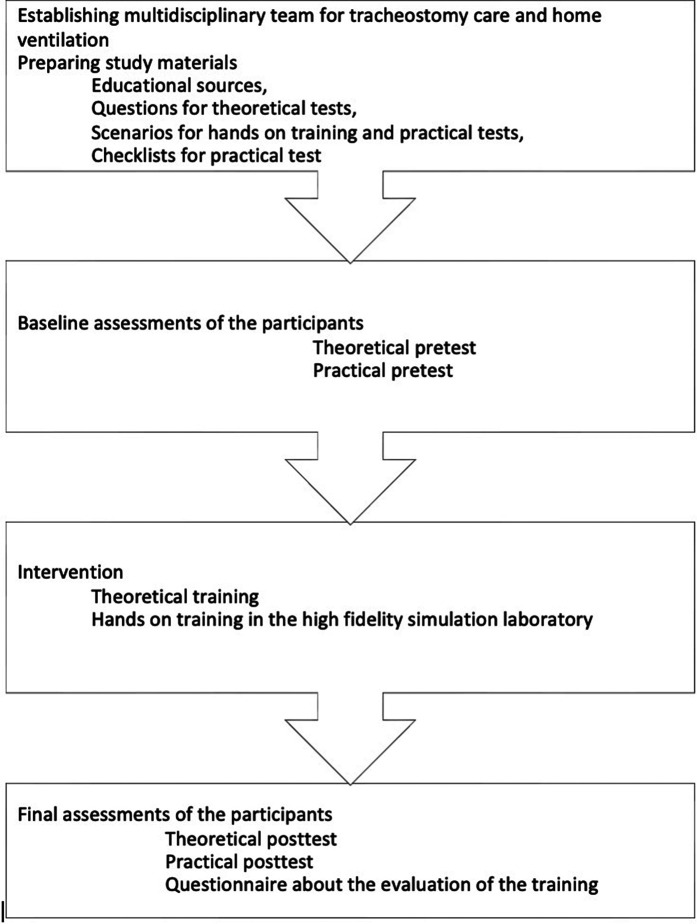
Study design.

At the end of the course, participants filled out an anonymous survey rating their experience on the elements of the training course by using a 4-point Likert scale (bad to excellent) (Figure 1).

### 2.5 Statistical analysis

Categorical variables were presented as frequencies and proportions. The outcome measurements included scores of the theoretical and practical pre-course and post-course tests. In the theoretical test, the number and percentage of participants who answered essential questions correctly and in the practical tests; the number and percentage of participants who performed the essential steps correctly were reported. Data were reported as mean ± SD for variables with normal distribution and median (25th–75th percentile) for variables which were not normally distributed. McNemar's test was used to compare the knowledge levels of the participants regarding essential questions. Wilcoxon test was used to compare continuous variables for two repeated measures. A *p*-value less than 0.05 was considered as statistically significant. Statistical analysis was conducted using SPSS version 22.0.

## 3 Results

The study included 43 physicians from 7 different tertiary centers of pediatric clinics where majority of children on LTIHV in Istanbul are followed. Among the participants, 46.5% (*n* = 20) were residents in pediatrics, 37.2% (*n* = 16) were fellows in pediatric pulmonology, 11.6% (*n* = 5) were fellows in pediatric intensive care/emergency department and 4.7% (*n* = 2) were pediatricians. Forty-one (95.4%) physicians were involved in the clinical follow-up of LTIHV patients. Twelve physicians (27.9%) stated to have good/excellent practical experience about equipment used in the care of the patients on home ventilation. Table 1 presents the baseline knowledge-skills of the physicians before training.

**Table 1:** Baseline knowledge and skills of the physicians before training.

Have you ever got a theoretical training about patients on LTIHV?	*n* (%)
Never	30 (69.8)
<6 months ago	3 (7)
6–12 months ago	2 (4.6)
>1 year ago	8 (18.6)
Have you ever got a practical training about patients on LTIHV?	
Never	27 (62.8)
<6 months ago	5 (11.6)
6–12 months ago	3 (7.0)
>1 year ago	8 (18.6)
Have you ever changed tracheostomy cannula?	
Never	4 (9.3)
I did an elective cannula change	12 (27.9)
I did an emergency cannula change	2 (4.7)
I did both	25 (58.1)
Have you ever got a training about basic life support?	
Never	4 (9.3)
<1 year ago	10 (23.3)
1–2 year ago	7 (16.3)
>2 year ago	22 (51.2)
Was the training adequate for the management of the urgent problems of the patients on LTIHV?	
No	37 (86.1)
Yes	6 (14.0)

LTIHV, long term invasive home ventilation.

Theoretical pre-test and post-test results are presented in Table 2. Post-training evaluation revealed a significant improvement in the percentage of correct answers in the theoretical test. The median (25th–75th percentiles) score increased from 26 (24–28) to 31 (29–32) (*p* < 0.001) (Table 2).

**Table 2:** Theoretical pre-test and post-test knowledge assessment in terms of the essential questions .

	Pre-test	Post-test	*p* value
Number of correct answers, median (25–75 p)	26 (24–28)	31 (29–32)	<0.001
Participants with correct answers to the essential questions *n*, (%)			
Recognition of signs of respiratory distress	36 (83.7)	42 (97.7)	0.03
Knowledge regarding basic life support	15 (34.9)	41 95.3)	<0.001
Knowledge of emergency bag content	37 (86)	43 (100)	0.03
Knowledge of the frequency of tracheostomy cannula change	36 (83.7)	42 (97.7)	0.03
Knowledge of correct tracheostomy cannula size in children	18 (41.9)	41 (95.3)	<0.001
Knowledge of proper suction technique	29 (67.4)	42 (97.7)	<0.001
Knowledge of proper duration of suction	26 (60.5)	42 (97.7)	<0.001
Indications for LTIHV	19 (44.2)	36 (83.7)	<0.001
Knowledge about ventilator equipment	27 (62.8)	40 (93)	0.001
Knowledge about ventilation modes	20 (46.5)	40 (93)	<0.001

LTHIV, long term invasive home ventilation.

**Figure 2:**
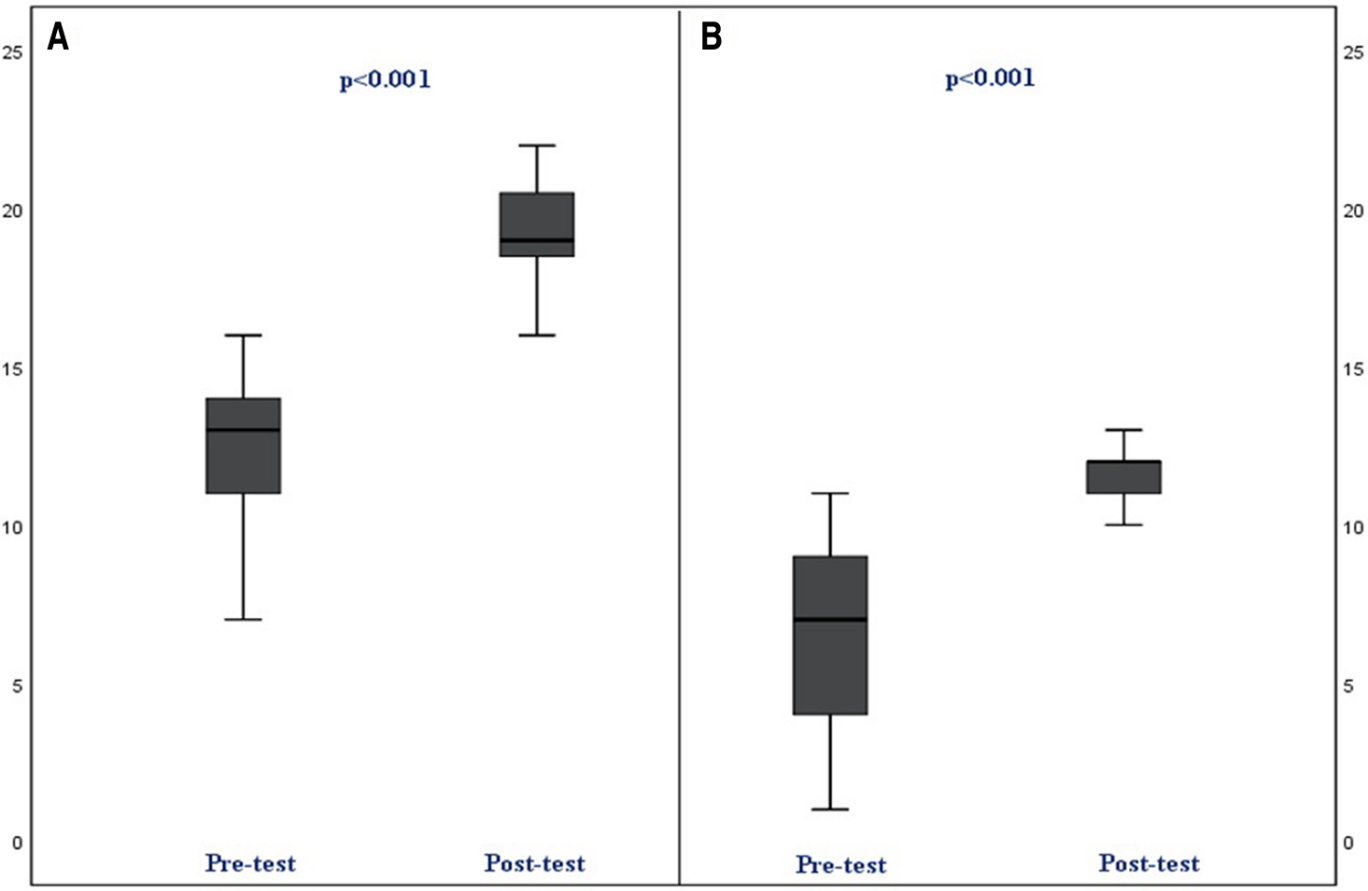
Pre-test and post-test assessment of the practical test for Scenario 1 and 2. (**A**) pre and post-test (including 22 steps) assessment for scenario (High pressure alarm). (**B**) pre and post-test (including 12 steps) assessment for scenario (Low pressure alarm).

In the practical test, the median number of correct steps after training increased significantly for both scenarios (*p* < 0.001, *p* < 0.001). Figure 2 shows the comparison of the results of the practical tests before and after the training. Pre-test and post-test assessment of the essential steps in the practical evaluation before and after training is presented in Table 3.

**Table 3:** Pre-test and post-test assessment of the essential steps in the practical evaluation before and after training.

Participants who performed the steps correctly (*n*, %)	Pre-test	Post-test	*p*-value
Scenario 1 (High pressure alarm)			
Checking the child	20 (46.5)	39 (90.7)	<0.001
Making a decision to aspirate	24 (55.8)	39 (90.7)	0.001
Suctioning the cannula	27 (62.8)	41 (95.3)	0.001
Changing the cannula	27 (62.8)	41 (95.3)	0.001
Scenario 2 (Low pressure alarm)			
Checking the child	24 (55.8)	40 (93)	<0.001
Connecting the ventilator to the test lung	10 (23.3)	38 (88.4)	<0.001
Checking the ventilator circuit and identifying the leak	17 (39.5)	42 (97.7)	<0.001
Changing the ventilator circuit	24 (55.8)	40 (93)	<0.001

After the training, participants were asked to evaluate their satisfaction with the course on a four-point Likert scale (bad to excellent). All of the 43 participants who responded rated the course overall as good or excellent.

## 4 Discussion

This multicenter study showed that a training program for physicians combining theoretical education and HFS training on tracheostomy and ventilator management led to significant improvements in the knowledge and practical skills of participants.

Children on LTHIV have a high risk of complications and adverse events, necessitating specialized professional and technical expertise for their care (17, 18). Although recent guidelines recommend education for physicians to acquire the necessary skills, there is no standardized curriculum and most physicians lack formal training (9, 14). Standardizing training of physician training can improve the outcomes of technology-dependent children (19). Physicians caring for these children must be able to recognize and handle ventilator emergencies in addition to managing tracheostomy-related complications. While studies have evaluated the effectiveness of training in airway emergencies and tracheostomy care, to our knowledge no study has assessed the effectiveness of training of physicians for ventilator emergencies in children with LTIHV (14, 16, 20, 21). Only one study has evaluated the effectiveness of an educational program on tracheostomy and ventilator emergencies for non-physician home care personnel (17). Ramsey et al. used a combination of theoretical and hands-on training and observed significant improvements in skill and comfort levels (17). In our study, 95% of the participants were involved in the care of LTIHV patients, however 85% stated that their training was inadequate for managing urgent problems. Lack of standard theoretical and practical training in tracheostomy care and LTIHV was observed in 70% and 63% of the physicians, respectively.

Advancements in the use of home ventilators allow patients to leave the pediatric intensive care unit earlier, and those on LTIHV frequently have high readmission rates after discharge (5, 22, 23). Most of these children can be safely cared in the wards which decreases hospital costs and increases the availability of intensive care units for more critical children, especially in low-resource settings like ours (24). Managing these technologically dependent children in the wards and at the outpatient clinics is challenging, even in developed countries, owing to a shortage of skilled healthcare providers (16, 25). Our study revealed a lack of knowledge and skills among physicians in managing children on LTIHV in Turkiye, highlighting the need for standardized training programs. After the training program, the number of correct answers in the theoretical post-tests increased significantly, with almost all participants answering questions about respiratory distress, basic life support, tracheostomy care, and mechanical ventilation correctly, indicating the effectiveness of the training.

HFS based training plays an essential role for teaching physicians about tracheostomy care and LTIHV emergencies, as real-life training may be limited owing to patient safety concerns and rareness of some emergency situations like cardiopulmonary arrest (26, 27). In the present study, immersive scenarios, such as ventilator high- and low-pressure alarms, home ventilator failure, and desaturation events requiring cardiopulmonary resuscitation, improved participants’ skills. During training, we emphasized assessing the patient's clinical status first, when a ventilator alarm was activated. Before training, only half of the participants controlled the patient in the first step in both scenarios, but this rate increased to nearly 100% after training. Human errors or inaudible alarms cause one-third of the reported adverse events associated with ventilator use (28). In a one-year study, 189 ventilator problems were reported in 150 adult and pediatric patients on LTIHV, with 30% due to mechanical failure, 30% due to improper usage by caregivers, and 3% due to misinterpreted changes in patients' clinical status (29). Following HFS based training, the participants' skills in recognizing ventilator alarms, determining their cause, and taking necessary actions significantly improved.

Ventilator malfunction and incorrect settings can lead to lung overdistention, hypoxia, hypoventilation, respiratory failure, or even death. Test lung is a ventilation bag that can be connected to the circuit to simulate resistance and compliance of the lungs. Using a test lung is helpful in quickly determining whether the problem is due to ventilator failure or a change in the patient's condition (30). In our study, we observed a significant increase in the use of the test lung from 23% to 88%.

Our study has several limitations. Assessments were made immediately after the study, so long-term outcomes are unknown. The participants were not selected randomly, volunteering of highly motivated physicians for the study might have influenced the results. Also, the members of the health care team other than the physicians were not included in the study, future studies involving nurses and practicing physicians are needed in order to determine the effectiveness of the course. The main strength of this study is the comprehensive program combining theoretical education and HFS based practical training.

In developed countries, typically family and professional caregivers provide care for children with LTIHV. However, inadequate training, medical device failure, and lack of caregiver vigilance (sleeping, alarm fatigue) are the main causes of preventable death in ventilator-dependent children at home. Proper training can increase caregiver confidence and readiness, and reduce readmission rates and death (31, 32). As home-care nursing is not readily available in Turkiye, in addition to educating physicians, education of parents who are responsible for caring for these children at home is crucial (33). Therefore, we also aim to implement a standardized training program for caregivers of children.

In conclusion, children with LTIHV have an increased risk of tracheostomy and ventilator related problems. Physicians require formal education. Our study demonstrated improved knowledge and skills through a combination of theoretical and practical training. Standardized educational programs can improve outcomes and reduce the healthcare burden.

## Data Availability

The original contributions presented in the study are included in the article/Supplementary Material, further inquiries can be directed to the corresponding author.
